# Implementing smoking cessation in routine primary care—a qualitative study

**DOI:** 10.3389/frhs.2023.1201447

**Published:** 2023-10-13

**Authors:** Petra Dannapfel, Preben Bendtsen, Marcus Bendtsen, Kristin Thomas

**Affiliations:** ^1^ Department of Health, Medicine and Caring Sciences, Linköping University, Linköping, Sweden; ^2^ Unit for Change Management and Support, Region Östergötland, Linköping, Sweden; ^3^ Department of Medical Specialists, Region Östergötland, Motala, Sweden

**Keywords:** smoking cessation, implementation science, telemedicine, primary health care, qualitative research

## Abstract

**Background:**

The use of digital tools has been proposed as a solution to some of the challenges of providing preventative services in primary care. Although there is a general acceptance among patients to use digital self-help tools to quit smoking, and healthcare organizations are increasingly urged to incorporate these tools in clinical practice, it is unclear how and for whom these innovations can be incorporated into clinical practice.

**Objectives:**

To explore health care professionals' perceptions about smoking cessation practice in routine primary care and the use of digital tools in this work.

**Methods:**

A qualitative study with nine in-depth telephone interviews with health care professionals working in primary care in Sweden. Convenience sampling and snowball technique was used as recruitment strategy. Informants included registered, district and auxiliary nurses as well as behavioral therapists. All informants were female, between 43 and 57 years old and experience of working with smoking cessation in primary care and possibility to recommend digital interventions to smokers.

**Results:**

Informants described smoking cessation practice in primary care as (i) identifying smoking patients, (ii) pursuing standardized routines for smoking cessation practice and (iii) keeping smoking cessation practice on the agenda. Digital tools were described by informants to be used in different ways: (i) replicating practice, (ii) complementing practice and (iii) enabling access to health care practitioners. Finally, the analysis showed that patients' expectations and behaviors contributed to how and when smoking cessation practice was conducted, including the use of digital tools.

**Conclusions:**

Implementing smoking cessation practice in primary care in Sweden encompass continuous work of reaching smoking patients, building buy-in among peers and keeping tobacco on the practice agenda. Digital interventions are used to replicate, complement and enabling access to care. The findings suggest that poor continuity of staff and negative attitudes towards preventative work may challenge smoking cessation practice. However, societal changes in the awareness of the health risks of tobacco use including shifting social norms regarding the acceptance of smoking may contribute to a normalization of speaking about smoking in primary care practice. Increased knowledge is needed on how, and for whom digital tools can be incorporated in clinical practice.

## 1. Introduction

In 2017, the Global Burden of Diseases, Injuries, and Risk Factors Study found that the second leading risk factor for disability adjusted life years was smoking (1), closely after high systolic blood pressure among the many factors considered. Smokers are at higher risk of contracting several non-communicable diseases, including cancer, diabetes, and cardiovascular and respiratory disease. Increasingly, research is also now showing that smokers could have an increased risk of severe COVID-19 (2, 3) and COVID-19 related mortality (4).

One of the key missions for primary health care is to work with health promotion and disease prevention, including screening for at-risk patients and offering support for smoking cessation. Various smoking cessation interventions have shown to be promising in supporting individuals to quit smoking for example, motivational interviewing and brief advice (5–7). The World Health Organization further argues that primary care is the most suitable setting for smoking cessation interventions and that professional support is needed to optimize results (8). However, routine screening for tobacco-use and systematically implementing smoking cessation interventions in a primary care context has proven difficult (9, 10). Barriers such as limited resources and heavy workload, lack of patient motivation for behavior change, poor has been cited to explain why preventative care is difficult to prioritize in primary care (11). Recent research confirms these findings but also suggest that limited training, negative perceptions about how patients can benefit from advice and thoughts on patient autonomy contribute to why primary care struggles to truly incorporate smoking cessation work in routine practice (12–14). In addition, research on the role of patients and their willingness to consider support shows that patient-related factors add to the mix and influence how health professionals approach patients' tobacco use during visits (15, 16).

The use of digital tools has been proposed as a solution to some of the problems of providing preventative services in primary care. Digital tools include those which are delivered to individuals via, for instance web platforms, mobile phone applications, and text messages. Evidence suggests that digital tools are effective in promoting a broad range of health behaviors, including smoking cessation (17–24). In addition, digital interventions have important potential given the opportunities to deliver relatively low-cost interventions at scale and the ubiquitous use of mobile phones in society irrespective of socioeconomic status (25). The content of digital smoking cessation interventions usually follows, or at least are consistent with interventions offered face-to-face including e.g., prompting and empowering the individual to make a quit attempt and then reinforcing and supporting this decision throughout the intervention period (23). Although there is a general acceptance among patients to use digital self-help tools to quit smoking, and healthcare organizations are increasingly urged to use these tools in clinical practice, more knowledge is needed on the willingness and capability of health care professionals to incorporate these innovations into clinical practice.

In an earlier study we have shown the effectiveness of a 12-week text message smoking cessation intervention (24). As the next step, a randomized trial implementation study was initiated to compare the effect on a 12-week text message smoking cessation intervention (the NEXit intervention) between smokers recruited in primary health care and smokers recruited online. This interview study was embedded within the above trial (26) and aimed to explore health care professionals' perceptions about smoking cessation practice in routine primary care and the use of digital tools in this work.

## 2. Methods

### 2.1. Study design

A qualitative study including nine in-depth individual telephone-interviews with health care professionals working at primary care centres in the south of Sweden. Data was analysed using content analysis according to Elo and Kyngäs (27). The consolidated criteria for reporting qualitative studies has been used in the writing of this manuscript and reporting on results (28).

### 2.2. Setting

This study was conducted at primary care centers in the south of Sweden. Primary care in Sweden is multi-professional and typically employs physicians, registered and specialized nurses as well as behavioral therapists. In addition, primary care centers can be connected to occupational health and physiotherapy services. Primary care is primarily responsible for health promotion, disease prevention and the treatment and management of illnesses, injuries, and long-term non-severe conditions.

### 2.3. Participants and data collection

Data collection was carried out between March 2021 and March 2022. Due to the COVID pandemic, health care professionals struggled to prioritize participating in interviews as it was even difficult to manage day-to-day practice and patient work. This was especially the case for those practices and time periods when centers carried out COVID vaccinations. Convenience sampling and snowball technique were therefore used in recruitment strategy. Initially, individuals meeting eligible criteria were invited via e-mail to take part. Snowballing was then added as a recruitment strategy whereby participants were asked in the end of interviews if they knew of a colleague that would be eligible to take part in interviews. Inclusion criteria were: (1) health care professionals working at a primary care center participating in the NEXit trial (26), (2) expected to engage in smoking cessation practice in the primary care context soon. Informants were recruited at primary health care centers that took part in the NEXit trial (26) to increase the likelihood of having experience of using digital tools in smoking cessation practice. A total of 31 eligible people were invited via e-mail and telephone to take part in interviews, 12 declined, ten did not respond, and a total of nine persons were thus interviewed. Informants were between 42 and 57 years of age, all women and included health promotion officers, registered nurses, specialized nurses (e.g., asthma), tobacco cessation specialists and district nurses (Table 1).

**Table 1:** Characteristics of the informants.

Informant	Age	Gender	Profession
1	43	Female	Health promotion officer
2	46	Female	Registered nurse
3	46	Female	Registered nurse, smoking cessation specialist and asthma/COPD
4	46	Female	Auxielly nurse and health promotion officer
5	60	Female	District nurse, smoking cessation specialist and asthma/COPD
6	53	Female	Health promotion officer
7	42	Female	Registered nurse, asthma/COPD
8	57	Female	District nurse, asthma/COPD
9	43	Female	District nurse

Written or verbal informed consent to take part in interviews was collected before interviews. A semi-structed interview guide was used and included questions on three themes: (i) current smoking cessation routines (e.g., can you describe how you initiate discussion about smoking with a patient?) (ii) screening of at-risk patients (e.g., can you tell me about how you currently work with smoking cessation practice at your clinic?”, (iii) experience of digital tools in smoking cessation practice and hopes for the future (e.g., can you tell me about your experiences of using mobile-phone based smoking cessation support in your clinical work?). The last author (KT, female behavior scientist with experience in health promotion research and qualitative methodology) conducted all the interviews via the telephone which lasted around 40 min however one interview was significantly shorter, 15 min. Field notes were taken after all the interviews to critically assess the content and use of the interview guide and procedures. Inductive thematic saturation was reached in that the emergence of novel codes ceased.

### 2.4. Data analysis

Data on the process of implementing smoking cessation counselling and the use of digital tools in routine practice was analyzed using content analysis according to Elo and Kyngäs (27). An inductive approach was employed and followed recommended steps: (1) the interview material was read a few times (KT and PD) to obtain a sense of the whole; (2) KT and PD then read all the interviews word by word to identify key data that could capture the implementation of smoking cessation practice and the use of digital tools in this work (i.e., open coding and the generation of coding sheets); (3) these codes were then labelled and (4) sorted (i.e., grouping) by KT and PD individually; KT and PD then discussed their groups of codes and potential categories (i.e., categorization). The remainder of the analysis process included discussions between KT and PD regarding the content of categories and contrasting content across categories (heterogeneity vs. homogeneity of categories) (i.e., categorization and abstraction). In this final phase, generic categories and subcategories were agreed upon.

## 3. Results

**Figure 1:**
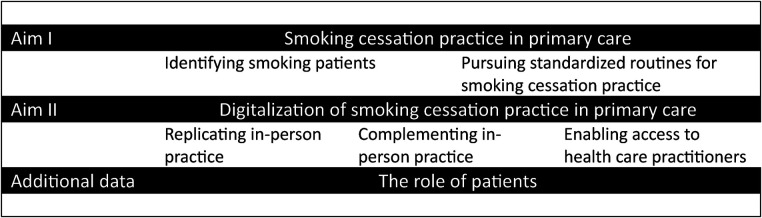
Overview of generic categories and sub-categories.

The analysis explored what smoking cessation practice entails in routine primary care in Sweden and what role digital tools play in this work. According to data, informants described smoking cessation practice in primary care as (i) identifying smoking patients, (ii) pursuing standardized routines for smoking cessation practice and (iii) keeping smoking cessation practice on the agenda. Furthermore, digital tools were described by informants to be used in different ways and purposes (i) replicating practice, (ii) complementing practice and (iii) enabling access to health care practitioners (Figure 1). Finally, the analysis showed that patients had a key role in how and when smoking cessation practice was conducted including the use of digital tools. Results from the analysis are described below including citations supporting the interpretation of data.

### 3.1. Smoking cessation practice in primary care

#### 3.1.1. Identifying smoking patients

Informants described that an important part of smoking cessation practice in primary care was to identify and reach at-risk patients i.e., smokers, that were willing to quit and that needed support. This work included screening for smoking during routinised health checks, screening and giving advice in general practice and primary prevention such as putting up information in waiting rooms. Informants talked about this work as mainly preventative and that it could be challenging to prioritize due to limited resources and poor buy-in among colleagues. This work required prioritizing the topic of tobacco which was especially difficult in general practice where patients typically came due to symptoms that were not clearly connected to their smoking. Informants also described multiple aspects of health that needed to be considered in general practice beyond what the patient sought care for e.g., domestic abuse and mental health concerns, which further challenged prioritizing preventative work.

In general, smoking was not perceived to be a sensitive subject. However, informants perceived that the extent to which patients were asked about smoking could depend on the interests and preferences of individual health care practitioners.

“… you see that it has to do with … interest and if you understand why we ask these questions about lifestyle. The nurses are more committed like, in general. And younger doctors I think are committed.” *Informant 1, female 43 years old health promotion officer*.

The data further suggested that the reach of smoking patients was influenced by the wider society and societal changes. For example, informants expressed that, changes of tobacco norms in society made their work easier as patients had an increased awareness and preparedness regarding the health risks of smoking and talking about tobacco in a health care situation. Informants compared smoking with talking to patients about alcohol which was believed to potentially be more sensitive and difficult to address. Furthermore, the COVID pandemic was perceived to have had influenced conditions with working preventative and patients' willingness to quit smoking. On one hand the pandemic had made it more difficult to manage preventative work due to tightened resources, on the other hand the pandemic raised the issue of the risk of tobacco and made patients more motivated to quit. In general, however, informants conveyed that they felt empowered and competent to ask about tobacco including knowing what to do if patients needed advice or referring to smoking cessation specialists.

“There has been a change. And it is more like, it is routine and so, that you talk about smoking and so, that you bring up that question.” *Informant 8, female 57 years old district nurse*.

#### 3.1.2. Pursuing standardized routines for smoking cessation practice

Smoking cessation practice was also described to involve working towards systematic and standardized routines regarding screening, referral, and treatment. For example, one informant expressed a vision that all patients coming through general practice in primary care would be asked about smoking and if they would be interested in support for quitting. Another informant stated that their unit already worked in this way whereby most patient visits in general practice addressed tobacco and smoking. For example, informants described how prompts in medical health records were used to remind health care professionals to ask about smoking during visits. Data in medical health records were also used to document rates of screening and treatment of smoking cessation at the units to be able to monitor the practice over time and feedback service outcomes to peers. Informants further described that standardized and formalized routines were achieved by formalizing roles and responsibilities for instance, allocating specialized nurses' explicit responsibility to receive referrals and follow-up ex-smokers. In general, the informants expressed that all these efforts were made to make smoking cessation practice part of the primary care structure and ways of working, making it less reliant on the motivation and preference of individual health care professionals. In addition, informants talked about the importance of access to specialized competency to be able to standardize smoking cessation practice. Most units where informants worked had access to certified smoking cessation specialists (e.g., nurses or counsellors) either on-site or within their county council. Furthermore, the patient work was described to follow a standardized program including continuous professional development such as peer-supervision and feedback.

“Yes, I think so. It's included in our routine questions, or what do you call it? In the medical records as prompts that you should sign. I think most of us do that. The maybe not every single time, if people come to visits often, but … but I think it is quite embedded to ask about tobacco.” *Informant 5, female 60 years old district nurse*.

### 3.2. Keeping smoking cessation practice on the agenda

Informants described a continuous, on-going process of building urgency among patients and peers and highlighted the importance of screening and treatment for smoking. Smoking cessation specialists were often described to facilitate smoking cessation practice and keeping the topic on the agenda. They were perceived to have a role in promoting smoking cessation practice among both patients and colleagues, and to some extent, be the link between the two. Informants mentioned other strategies that were used in building urgency such as adding smoking cessation to agendas for management and steering groups, monitoring and following-up practice rates on e.g., the number of smokers identified and helped to quit smoking. For example, one informant described how nurses specialized in smoking cessation had been included in the steering group at the center which was perceived to legitimize the topic of tobacco at their workplace. The informants highlighted factors relating to resources and organization that made it more difficult to achieve a continuation in smoking cessation practice. For example, difficulties in hiring permanent staff were described by one informant as something that contributed to making smoking cessation practice arbitrary.

“Yes. It varies also. Because it is difficult to get permanent doctors hear, // So we have like a high turnover. It came, we hire temporary doctors and so. And it is a huge difference in how they work. Some of them work a lot, then I get many referrals. As soon as they [patients] say they smoke they get referred to me for smoking cessation advice, even if they are not ready to quit. And others don't ask about tobacco at all. So, it varies.” *Informant 4, female, 46 years old auxiliary nurse and health promotion officer*.

### 3.3. Digitalization of smoking cessation practice in primary care

The data showed that digital tools were used in smoking cessation support (patient work) in different ways and for different reasons specifically in (i) replicating practice, (ii) complementing practice and (iii) enabling access to health care practitioners. Informants talked about a variety of digital tools: online meetings, interventions delivered via mobile phones (applications and text messaging) that were automated or used manually by personnel, as well as internet-based interventions. The COVID-19 pandemic was perceived to have contributed to an increased use of digital tools as a response to restrictions and patient preferences. For example, patients that found that meeting in person was too much, were offered digital tools and referral to a smoking cessation app.

#### 3.2.1. Replicating in-person practice

The use of digital tools involved digitally replicating practice that was otherwise performed in-person, through for example online meeting tools or mobile applications. This way of using digital tools, for example online meetings with patients, were especially described to have increased during the COVID-19 pandemic and were thought to be comparable with in-person interventions in terms of perceived opportunities and challenges. One informant perceived that both patients and health care professionals have become more confident and experienced during the pandemic to use digital tools in health care situations. Some informants acknowledged that patients preferred meeting online or receiving mobile-phone based support and could thus see great potential in using digital tools in their practice. Informants also mentioned challenges with using digital tools in this way and expressed that they preferred meeting patients in-person when they could gauge patients' needs and situation better and accommodate accordingly. When using digital tools in this way, informants highlighted that the content of practice, the support given, the approach employed, were consistent with the patient work that would have been performed in-person e.g., motivational interviewing techniques.

“Yes, it really doesn't differ from meetings in-person, other than that we meet remotely online like. Via a secure link that is provided by the primary care, yeah that all centres can use.” *Informant 1, female 43 years old health promotion officer*.

#### 3.2.2. Complementing in-person practice

A variety of digital tools were used as a complement to in-person delivered smoking cessation support, for instance mobile applications and text messaging interventions. These tools were primarily targeting patients directly, using automated one-way communication. These tools were primarily recommended for patients and used as a complement to standard care as they offered support also in-between care visits. These tools were also used as a complement for those patients that were not committed to attending smoking cessation counselling sessions at the clinic. Thus, these tools were described to involve minimum effort and interaction from the health care professional. For instance, informants explained that limited, or no follow-up, was usually done by the clinics regarding how patients perceived the support given in the tools, or regarding the effectiveness of the tools. Also, some informants expressed that they were not comfortable in recommending specific tools, but rather, that they left this choice up to the patient to decide. The interviews suggest that a reason for this passive approach may be due to that informants see a limitation with certain digital tools, that they are difficult to tailor and make interactive and that informants perceived that digital tools such as mobile applications are not suitable for all patients. Informants also described that using digital tools in this way, as a complement, was depending on the preference and interest of the health care professional, and the patient.

“While the support that they get from me during visits is more personal and adapted to that specific person's problems and challenges. Maybe some situations that are difficult to solve, and we want to look at, how can we solve this? What can I do instead? And so on. That you have to discuss and ventilate about stuff that feels tough. And… That maybe you don't get from an app, I think.” *Informant 3, female 46 years old registered nurse*.

#### 3.2.3. Enabling access to health care practitioners

Digital tools were also used to enable access to health care practitioners by communicating with patients via chats or online interface. Digital tools were used for this purpose to offer support in-between visits, reach certain patient groups and when resources were scarce. For example, one informant described that they used a mobile application which offered continuous support in-between visits but also allowed patient-practitioner interaction via a message-function and the ability for practitioner to tailor content. Interactive tools were also used to increase access to health care practitioners among patients that did not speak Swedish. One informant described that although they had access to interpreters, they perceived that using digital tools such as mobile applications was preferred among patients. Informants described interactive tools as more demanding due to expectations on being available outside of booked visits.

“Yes, for me it is like that, but it depends how you plan your work, it is [laughter], that you become, can become stressed. If you have like booked in patients that are coming here, then they have their booked time. Hear [online support tool] I get that they nudge me and require my attention all the time. Because I get e-mails when they have been logged on and written something. Which can be a bit stressful for me.” *Informant 4, female 46 years old auxiliary nurse and health promotion officer*.

### 3.4. The role of patients

The interviews showed that patients had a key role in how and when smoking cessation practice was conducted, including the use of digital tools. In general, informants expressed that multiple and a variety of methods are needed to reach smokers and to help patients quit. Informants described that a patient-centered approach was needed in their work whereby content and communication was adapted and accommodated to the needs and preferences of individual patients. The choice and use of digital tools followed this manner and were as much as possible tailored to the situation. Informants described patients as “gatekeepers” as health care practitioners gauged patients' motivation, engagement, commitment for change and accommodated approach accordingly. For example, if a patient expressed limited motivation to quit, they were not referred to a specialist which would require commitment and effort from the patient. However, the less motivated patient could be recommended an app (automated).

Patients were also described to adopt active roles by seeking care themselves and pushing for certain treatments or medications. Furthermore, one informant described that patients' expectations on the role of health care and the relationship between patient-practitioner, that sometimes are connected to culture, need to be considered in this work. For example, smoking cessation work was described as more challenging in-patient groups living in cultures where tobacco use was more socially accepted. The fact that patients play such a central role in the implementation of both smoking cessation practice and the use of digital tools, was perceived by informants to make the work unpredictable. Informants described that it was difficult to know whether a patient would stick to a behaviour change, use a specific app or whether patients would respond to follow-up phone calls.

“… Yes, but it is different for different people, because sometimes I think that this person will quite, this person won't quit. And then they quit. And you can't say what did it. And you have given them the same information like. So, it is really difficult to tell sometimes.” *Informant 2, female 46 years registered nurse*.

## 4. Discussion

This study explored perceptions among health care practitioners regarding smoking cessation practice in primary care and the use of digital tools in this work using in-depth interviews. The analysis showed that smoking cessation practice involves striving to identify at-risk smoking patients, working towards standardized routines, and continuously building urgency for the practice and putting the topic on the agenda for primary care. The analysis regarding the use of digital tools showed that a variety of tools were used to replicate practice, complement practice, and enable access to cessation support. Having different tools to choose between could give the practitioner more opportunities for tailored support. A tool ensuring patient preferences are more likely to support and trigger the behavior in the desired direction. The data suggested that practitioners predominantly worked with, and chose, digital interventions in a pragmatic way to fit patients' preferences.

The findings showed that patients have a central role in smoking cessation practice and the use of digital tools. One informant even described patients as gatekeepers for the implementation of tobacco work which relied on patients' motivation and commitment to quit, but also to the use of digital tools. This is in line with most clinical guidelines and national recommendations on how to work with smoking cessation and tobacco control in primary care (29, 30). Although most guidelines recommend a variety of methods (e.g., 5A approach, motivational interviewing, or brief advice), guidelines primarily cover patients that are motivated to quit, thus patients that already exhibit a level of readiness for change (29, 30). Our findings showed that digital tools were somewhat used among patients that expressed poor motivation to quit smoking. In these situations, digital solutions were primarily used as a complement to in-person support. A reason for this secondary use of digital tools could be that Swedish national guidelines for disease prevention and health promotion (including tobacco control) do not explicitly recommend automated digital interventions (29, 30). Although web-based interventions are included in national guidelines, interactive and tailored support (online or in-person) is advised including gauging patients' readiness for change. Similarly, the evidence-base for digital smoking cessation interventions (e.g., mobile applications and text messaging) can mostly be found among smokers reporting high readiness to quit (22, 31, 32). Health care organizations, and perhaps especially the primary health care setting, are important arenas for reaching non-motivated smokers in terms of both health education and offering smoking cessation support. However, to reach these individuals, evidence-based interventions need to be fully implemented and incorporated in everyday routines.

We know from the implementation research field that facilitation and effective implementation strategies are often needed to succeed with implementation long-term (33, 34). Several strategies can be found that support health care practitioners to perform smoking cessation activities and cue their behavior in the “right” direction. One way to maximize support for self-regulation is to change routines and environment (35). The use of prompts in medical health records reminds peers to screen and refer patients that might be willing to quit smoking. Prompts help to engage in and perform activities needed to reach patients and thus their opportunities be involved in smoking cessation. Our findings showed that various strategies were used to push for smoking cessation practice being prioritized at the centers. For example, standardized routines such as using medical records to record smoking behavior were used to compensate for potential individual differences among practitioners in how often patients were asked about smoking. Thus, this was an attempt to bypass low engagement or negative attitudes towards prioritizing tobacco among clinicians and prompting behavior explicitly. To incorporate smoking cessation practice within routines long-term however, clinical behaviors such as tobacco screening in general practice would require acceptance among practitioners including clinical behavior change. Michie et al. (35) emphasize the importance of specifying target behaviors in detail and then identify what needs to be done differently in order to achieve the target behavior. The outcome of implementing smoking cessation is to help patients quit smoking. However, to reach that goal health care practitioners need to perform certain behaviors for instance screening, referral, choose the right treatment and then support and guide patients through the treatment. In our findings, the prevalent barriers for implementing smoking cessation practice in routines were poor buy-in among practitioners and lack of continuity due to temporary staffing. In addition, the findings suggested that challenges in incorporating smoking cessation in practice were due to the preventative nature of the topic, rather than the topic itself. A study including interview data with practitioners and patients showed a resistance among practitioners towards preventative tasks in smoking cessation practice and positive attitudes towards e.g., prescribing medication for smoking cessation (14). Thus, to further reinforce smoking cessation practice in primary care, the importance of preventative work *per se* may need to be emphasized. Furthermore, our data highlights temporary staffing as a key barrier which suggests that preventative work is not necessarily part of the primary care culture, but rather, relies on specific individuals or professions to reinforce implementation making it difficult to prioritize during times of high staff turnover and scarce resources.

Furthermore, there are a series of collective factors that make smoking cessation practice incorporated in routines. Indeed, implementing smoking cessation involves collaborative work among peers as multiple health care professionals are needed to achieve for instance in screening, smoking cessation advice and support as well as long-term follow-up of behavior change. Michie et al. (35) state that behaviors occur within a context of other behaviors and are thus part of, and interacts, with a larger system (in this case the health care team and primary care center). Implementing smoking cessation in primary care needs collaborative work that involves the investment of both personal and group resources to achieve the goal. Having a designated health care practitioner as a smoking cessation specialist with the responsibility to promote smoking cessation activities among colleagues will likely contribute to the implementation of smoking cessation to patients. Designated roles and responsibility can be considered key resources, reminding peers about the importance of asking patients about smoking, cascading training and getting preventive work on the agenda. Having advocates that are knowledgably, committed, and active have shown to positive impact implementation outcomes in interventions (36). They are also a practical support and modelling behavior to get peers involved in smoking cessation.

Our findings also contradict some earlier research. A systematic review and meta-synthesis of qualitative research on patients' and practitioners’ lived experience of tobacco use and smoking cessation practice (37) highlights a general experience of lack of legitimacy among practitioners regarding addressing smoking during patient visits. Specifically, the review, that included 22 studies, reports that practitioners expressed a lack of sincerity and adequacy as well as perceived lack of skills and training regarding smoking cessation practice. On the contrary, in our interviews, practitioners expressed confidence and self-efficacy in asking patients about smoking and offering advice. Our data further showed that tobacco as a topic was not perceived to be sensitive or difficult to initiate during patient visits. These inconsistent findings could be explained by the increased awareness of the risk of smoking that has occurred in the last decade in society, making tobacco relevant in general practice both from patients' and practitioners' perspectives. Indeed, the studies in the above review (37) referring to limited self-efficacy are not recent e.g., Heath et al. (38) and Kerr et al. (39) suggesting that the place for tobacco in primacy care may have become normalized.

### 4.1. Methodological considerations

Due to the COVID pandemic, the period of data collection had to be adapted to conditions and resources at the primary care centers. The interviews were carried out over a rather long period of 12 months which could have influenced the findings. However, the research questions aimed to capture topics that we did not deem to be time-sensitive (at least not changeable over a period of a year) for example, perceptions about the role of smoking cessation in routine practice. If anything, the longer period of data collection could have contributed to richer data on smoking cessation practice.

Trustworthiness of the study procedures was considered in numerous ways (40, 41). Truth value of the data (credibility) was achieved by employing methods as systematically as possible and investigator triangulation in the analysis process. In addition, the prolonged data collection can have increased the truth value of the material. However, credibility could have been further increased by a broader informant group, also including for instance male participants or physicians to gain a richer picture of smoking cessation practice in routine primary care. Another limitation of the study is the potential bias in sampling as taking part in interviews, due to the COVID situation, was difficult to prioritize for all. Consistency in study procedures (dependability) was increased using the interview guide and field notes which encouraged the interviewer to continuously assess both the guide and specific interview situations. Finally, to increase the applicability of the findings (transferability) has primarily been addressed by following COREQ guidelines in reporting (28).

### 4.2. Conclusions

Implementing smoking cessation practice in primary care in Sweden encompass continuous work of reaching smoking patients, building buy-in among peers and keeping tobacco on the practice agenda. Digital interventions are used to replicate, complement and enabling access to care. The findings suggest that poor continuity of staff and negative attitudes towards preventative work may challenge smoking cessation practice. However, societal changes in the awareness of the health risks of smoking including shifting social norms regarding the acceptance of smoking may contribute to a normalization of speaking about smoking in primary care practice.

## Data Availability

The raw data supporting the conclusions of this article will be made available by the authors, within reasonable request.
